# Viabahn stent graft for arterial injury management: safety, technical success, and long-term outcome

**DOI:** 10.1186/s42155-024-00435-9

**Published:** 2024-02-28

**Authors:** Jan M. Brendel, Tobias Mangold, Mario Lescan, Jörg Schmehl, Patrick Ghibes, Antonia Grimm, Simon Greulich, Patrick Krumm, Christoph Artzner, Gerd Grözinger, Arne Estler

**Affiliations:** 1 Department of Radiology, Diagnostic and Interventional Radiology, Tübingen University Hospital, Hoppe-Seyler-Straße 3, Tübingen, 72076 Germany; 2 Department of Anesthesiology and Intensive Care Medicine, Tübingen University Hospital, Tübingen, Germany; 3 Department of Thoracic and Cardiovascular Surgery, Tübingen University Hospital, Tübingen, Germany; 4 Department of Cardiology and Angiology, Tübingen University Hospital, Tübingen, Germany; 5 Department of Radiology, Diakonie Klinikum, Stuttgart, Germany

**Keywords:** Viabahn, Endoprosthesis, Stent, Graft, Injury, Bleeding, Heparin

## Abstract

**Background:**

The Viabahn stent graft has emerged as an integral tool for managing vascular diseases, but there is limited long-term data on its performance in emergency endovascular treatment. This study aimed to assess safety, technical success, and long-term efficacy of the Viabahn stent graft in emergency treatment of arterial injury.

**Methods:**

We conducted a retrospective single tertiary centre analysis of patients who underwent Viabahn emergency arterial injury treatment between 2015 and 2020. Indication, intraoperative complications, technical and clinical success, and major adverse events at 30 days were evaluated. Secondary efficacy endpoints were the primary and secondary patency rates assessed by Kaplan–Meier analysis.

**Results:**

Forty patients (71 ± 13 years, 19 women) were analyzed. Indications for Viabahn emergency treatment were extravasation (65.0%), arterio-venous fistula (22.5%), pseudoaneurysm (10.0%), and arterio-ureteral fistula (2.5%). No intraoperative adverse events occurred, technical and clinical success rates were 100%. One acute stent graft occlusion occurred in the popliteal artery on day 9, resulting in a 30-day device-related major-adverse-event rate of 2.5%. Median follow-up was 402 days [IQR, 43–1093]. Primary patency rate was 97% (95% CI: 94–100) in year 1, and 92% (95% CI: 86–98) from years 2 to 6. One stent graft occlusion occurred in the external iliac artery at 18 months; successful revascularization resulted in secondary patency rates of 97% (95% CI: 94–100) from years 1 to 6.

**Conclusion:**

Using Viabahn stent graft in emergency arterial injury treatment had 100% technical and clinical success rates, a low 30-day major-adverse-event rate of 2.5%, and excellent long-term patency rates.

## Background

The heparin-bonded, self-expanding polytetrafluoroethylene (ePTFE) covered Viabahn endoprosthesis is primarily renowned as an effective alternative to conventional open bypass surgery for the treatment of peripheral artery disease [1–3]. However, the Viabahn stent graft is increasingly being used for emergency endovascular therapy (EVT) of arterial injuries [4, 5]. Outcomes resulting from arterial bleeding can vary in severity from minor to life-threatening, and depend on various factors including the size and location of the injured artery, the speed of medical intervention, and the overall health of the individual. Adverse consequences of arterial bleeding may comprise hemorrhagic shock, organ damage, infection, and loss of limb function. Given the severity and complexity of arterial bleeding, prompt and proper medical treatment is crucial to minimize the risk of adverse outcomes. Such cases present unique challenges and often require endovascular treatment. An established device is the Viabahn endoprosthesis. These flexible endoprostheses provide precise delivery capability, rapid hemostasis and structural support, making them ideal for managing intricate and complex lesions, and for deploying in small or anatomically challenging vessels [6, 7]. Despite the increasing use of Viabahn endoprosthesis in the setting of emergency EVT, comprehensive data on its long-term performance encompassing multiple indications remain scarce [8–10].

However, data on its long-term performance is crucial for the treating physician as it has the potential to provide a deeper understanding of the device's safety and efficacy, improve indication and ultimately patient care. An all-comers approach seems reasonable, as many endovascular devices have shown decreased real-life performance compared to clinical trial results [11, 12]. Therefore, this study aims to evaluate safety, technical success, and long-term efficacy of heparin-bonded Viabahn stent grafts in emergency EVT for arterial vessel injuries.

## Methods

### Patient population

A tertiary single-center (Tübingen university hospital, Tübingen, Germany) study consecutively recruited and retrospectively analyzed patients who underwent emergency EVT using the Viabahn stent graft. An all-comers approach was chosen, enrolling patients regardless of their condition. Adult patients (age ≥ 18 years) undergoing clinically indicated emergency Viabahn procedure due to vascular injury between March 2015 and September 2020 were included. Patient baseline characteristics were recorded, including obesity, arterial hypertension, diabetes mellitus classified as non-insulin-dependent (NIDDM) or insulin-dependent (IDDM), dyslipidemia, family history of premature coronary artery disease, and smoking status classified as current or former smoker and never smoked. Comorbidities were noted, including chronic obstructive pulmonary disease (COPD), cerebrovascular or coronary artery disease, and atrial fibrillation. Renal function was assessed by laboratory measurement of glomerular filtration rate (GFR), and the presence of chronic renal failure (GFR < 60 mL/min/1.73m^2^) or current hemodialysis was recorded. Evidence of bacteremia and known uncorrectable hypercoagulability were noted. The study was approved by the institutional review board (IRB approval number: 254/2021BO1, date: 2021/08/20) and adhered to the regulations of the Health Insurance Portability and Accountability Act (HIPAA). Informed consent was waived by the IRB due to the nature of the study.

### Study procedure

EVT was conducted using a 4F sheath and a common femoral or brachial artery access, with either local anesthesia and conscious sedation or general anesthesia. A catheter was then advanced to the target artery's distal side with the support of a guidewire. Target artery angiography was performed to display any bleeding, and the guidewire was substituted with a stiff wire to replace the sheath with a dedicated 6F to 12F sheath. The sheath was advanced to the distal side or as close to the intended target artery as possible. For stent graft delivery, a 0.018-inch or 0.035-inch stiff wire was utilized. The Viabahn endoprosthesis (5–13 mm in diameter; 2.5–25.0 cm in length) was then inserted and positioned over the site of the intended target artery for deployment. The initial Viabahn procedure's characteristics were assessed, including the target vessel, number of stents used, the diameter (mm) and length (cm) of the stent graft, and neck length (in mm, defined as the minimum distance between the edge of the stent graft and the affected or bleeding site). Angiography was immediately carried out after the deployment. After confirming successful stent placement with full coverage of the bleeding site, Heparin (5,000 IU) was administered for anticoagulation. Closure of vascular access sites was performed either through manual closure by local compression (6F) or applying a vascular closure device (≥ 6F). Pre-closing was performed in lager-sized sheaths > 8F. After the procedure, coagulation optimization and, in some cases, eptifibatide bridging were recommended. When the patient's vital signs were stable and no other contraindications were present, anticoagulation therapy was prescribed based on dual antiplatelet therapy consisting of aspirin (100 mg/day) and clopidogrel (75 mg/day) for at least 6 months to prevent stent graft thrombosis. Subsequently, single antiplatelet therapy with aspirin (100 mg/day) was prescribed for lifetime.

### Assessment of EVT efficacy and follow-up

Acute technical success rate was evaluated, based on pre-established criteria: completed placement of Viabahn stent graft with full coverage and exclusion of the injured vessel area, and preservation of blood flow in the artery, as confirmed by final target site angiography. Clinical success was deemed complete hemostasis within 30 days post-procedure. Prior to hospital discharge, all patients received CTA or ultrasound examinations to ensure sufficient blood flow. A protocol for follow-up care was recommended following the index procedure, with scheduled visits for physical examination and duplex ultrasound at 30-day, 3-month, 6-month, and 12-month intervals, followed by annual visits.

### Study endpoints

The primary safety endpoint was freedom from major adverse events (MAE) at 30 days, including acute thrombotic occlusion, major target limb amputation, myocardial infarction, and death. The secondary efficacy endpoint was the primary and secondary patency rate. In the case of stent graft stenosis/occlusion, time (days) after initial procedure and type of clinically driven target lesion revascularization (cdTLR: surgical or endovascular procedure) was recorded.

### Statistical analysis

Normality of data was verified using the Shapiro–Wilk test. Continuous data are presented as mean ± standard deviation for parametric data or as median [IQR] for non-parametric data; categorical data are given as counts (percentages). Survival curves (primary and secondary patency) were estimated using the Kaplan–Meier method, SPSS version 29.0, IBM.

## Results

### Patient characteristics

A total of 40 patients (mean age 71 ± 13 years, 21 men, 19 women) who received emergency EVT using Viabahn stent graft were included and analyzed in this study. Baseline patient characteristics are shown in Table 1. Most common cardiovascular risk factor was dyslipidemia (13/40, 32.5%), followed by diabetes mellitus (10/40, 25.0% with NIDDM and 2/40, 5.0% with IDDM), arterial hypertension (10/40, 25.0%), and obesity (10/40, 25.0%). Most frequent comorbidity was coronary artery disease in 10/40 patients (25.0%), followed by atrial fibrillation in 8/40 (20.0%), chronic obstructive pulmonary disease (COPD) in 7/40 patients (17.5%), and cerebrovascular artery disease in 2/40 patients (5.0%). Median GFR was 77 [IQR, 35–118] mL/min/1.73m^2^, chronic renal failure was observed in 11/40 patients (27.5%), and there were no patients on hemodialysis. There was no evidence of bacteremia in any patient.

**Table 1:** Baseline patient characteristics

Parameter	Total no. of patients *n* = 40
Age	
at intervention, years	71 ± 13
range, years	34–92
< 65 years	11/40 (27.5)
65–75 years	7/40 (17.5)
> 75 years	22/40 (55.0)
Sex at birth	
Male	21/40 (52.5)
Female	19/40 (47.5)
Body mass index, kg/m^2^	28 ± 7
Cardiovascular risk factors	
Obesity	10/40 (25.0)
Arterial hypertension	10/40 (25.0)
Diabetes	
no	28/40 (70.0)
NIDDM	10/40 (25.0)
IDDM	2/40 (5.0)
Dyslipidemia	13/40 (32.5)
Family history of CAD	2/40 (5.0)
Smoking status	
Current smoker	3/40 (7.5)
Former	8/40 (20.0)
Never	29/40 (72.5)
Comorbidities	
COPD	7/40 (17.5)
Cerebrovascular artery disease	2/40 (5.0)
Coronary artery disease	10/40 (25.0)
Atrial fibrillation	8/40 (20.0)
Renal function	
GFR (mL/min/1.73m^2^)	77 [35–118]
Chronic renal failure (GFR < 60)	11/40 (27.5)
Hemodialysis	0/40 (0.0)
Known uncorrectable hypercoagulability	0/40 (0.0)
Evidence of bacteremia	0/40 (0.0)

Values are mean ± SD, median [IQR] or frequency n (%)

*NIDDM* non-insulin-dependent diabetes mellitus, *IDDM* insulin-dependent diabetes mellitus, *CAD* coronary artery disease, *COPD* chronic obstructive pulmonary disease, *GFR* glomerular filtration rate

### Indication and target vessels

The indications for EVT in this study were active uncontained hemorrhage (extravasation) in 26/40 patients (65.0%), arterio-venous fistula in 9/40 patients (22.5%), pseudoaneurysm formation resulting from contained hemorrhage in 4/40 patients (10.0%), and arterio-ureteral fistula in 1/40 patients (2.5%), Table 2. None of the study patients had upper extremity arterial bleeding. In the abdomen, the celiac trunk was targeted in 1/40 patients (2.5%), the hepatic artery in 2/40 patients (5.0%). In the pelvis, the most targeted vessel was the external iliac artery (8/40, 20.0%), followed by the internal iliac artery (2/40, 5.0%). In the lower extremity, the most targeted vessel was the superficial femoral artery (16/40, 40.0%), followed by the common femoral artery (6/40, 15.0%), and the popliteal artery (5/40, 12.5%).

**Table 2:** Indication and target vessels

Parameter	Total no. of patients *n* = 40
Indication	
Extravasation	26/40 (65.0)
Arterio-venous fistula	9/40 (22.5)
Pseudoaneurysm	4/40 (10.0)
Arterio-ureteral fistula	1/40 (2.5)
Target vessel	
Upper extremity	0/40 (0.0)
Abdomen	
Celiac trunk	1/40 (2.5)
Hepatic artery	2/40 (5.0)
Pelvis	
Common iliac artery	0/40 (0.0)
External iliac artery	8/40 (20.0)
Internal iliac artery	2/40 (5.0)
Lower extremity	
Common femoral artery	6/40 (15.0)
Superficial femoral artery	16/40 (40.0)
Deep femoral artery	0/40 (0.0)
Popliteal artery	5/40 (12.5)

Values are given as frequency (%)

### Stent graft procedure characteristics

EVT was performed via either common femoral (39/40, 97.5%) or brachial (1/40, 2.5%) artery access, Table 3. In total, 43 stent grafts were deployed, with the most commonly used device diameter being 6 mm (15/43, 34.9%), followed by 8 mm (9/43, 20.9%), 7 mm (8/43, 18.6%), and 5 mm (7/43, 16.3%). Diameters > 8 mm were deployed in 4/43 cases (9.3%). The most frequently used stent graft length was 5.0 cm (17/43, 39.5%), followed by 10.0 cm (14/43, 32.6%) and 2.5 cm (8/43, 18.6%). Devices of 15.0 cm and 25.0 cm were utilized in 3/43 cases (7.0%) and 1/43 cases (2.3%), respectively. The median neck length was 20 mm [IQR, 12–30]. Closure devices were employed in 35 of 40 cases (87.5%); we predominantly used FemoSeal (17/40, 42.5%), followed by StarClose (8/40, 20%), ProGlide (5/40, 12.5%), and MynxGrip (5/40, 12.5%). No intraoperative adverse events occurred, and all 40/40 procedures (100%) had technical success.

**Table 3:** Procedure characteristics

Parameter	Total no. of patients *n* = 40
	Total no. of deployed devices *n* = 43
Approach site	
Femoral	39/40 (97.5)
Brachial	1/40 (2.5)
No. of devices implanted	
1	38/40 (95.0)
2	1/40 (2.5)
3	1/40 (2.5)
Device diameter (n, % of 43 deployed)	
5 mm	7/43 (16.3)
6 mm	15/43 (34.9)
7 mm	8/43 (18.6)
8 mm	9/43 (20.9)
> 8 mm	4/43 (9.3)
Device length (n, % of 43 deployed)	
2.5 cm	8/43 (18.6)
5.0 cm	17/43 (39.5)
7.5 cm	0/43 (0.0)
10.0 cm	14/43 (32.6)
15.0 cm	3/43 (7.0)
25.0 cm	1/43 (2.3)
Neck length (mm)	20 [12–30]
Intraoperative adverse events	0/40 (0.0)
Technical success^a^	40/40 (100)

Values are given as frequency n (%) or median [IQR]

^a^Technical success was defined as the completed placement of the Viabahn stent graft with full bridging of the vessel injury, as determined by final target site angiography

### Acute and 30-day outcomes

40/40 procedures (100%) had clinical success, Table 4. Device- or procedure-related death, myocardial infarction, or major amputation occurred in no patient (0/40 patients) within 30 days of stent graft placement. Acute thrombotic occlusion was observed in the popliteal artery of 1 out of 40 patients (2.5%): complete thrombectomy was not successful, and temporary lysis therapy was required. During re-intervention, rupture in the P2 segment with a residual thrombus was detected. A subsequent Viabahn implantation resulted in successful recanalization. At the 30-day mark, 97.5% of the patients (39/40) were free of any major adverse effects.

**Table 4:** Clinical and procedural outcomes

Parameter	Total no. of patients *n* = 40
Clinical success^a^	40/40 (100)
MAE within 30 days	
Death^b^	0/40 (0.0)
Myocardial infarction	0/40 (0.0)
Major amputation	0/40 (0.0)
Acute thrombotic occlusion	1/40 (2.5)
Primary patency	
3 months	97 (94–100)
6 months	97 (94–100)
1 year	97 (94–100)
2 years	92 (86–98)
3 years	92 (86–98)
4 years	92 (86–98)
5 years	92 (86–98)
6 years	92 (86–98)
Secondary patency	
3 months	97 (94–100)
6 months	97 (94–100)
1 year	97 (94–100)
2 years	97 (94–100)
3 years	97 (94–100)
4 years	97 (94–100)
5 years	97 (94–100)
6 years	97 (94–100)
cdTLR	
Total	2/40 (5.0)
Endovascular treatment	2/40 (5.0)
Surgical treatment	0/40 (0.0)
Major amputation	0/40 (0.0)
Minor amputation	0/40 (0.0)

Values are given as frequency (%) or percent (95% CI)

*MAE* major adverse event, *cdTLR* clinically driven target lesion revascularization

^a^Clinical success was defined as complete hemostasis within 30 days of the procedure

^b^Death, device- or procedure-related

### Long-term outcomes

**Fig. 1:**
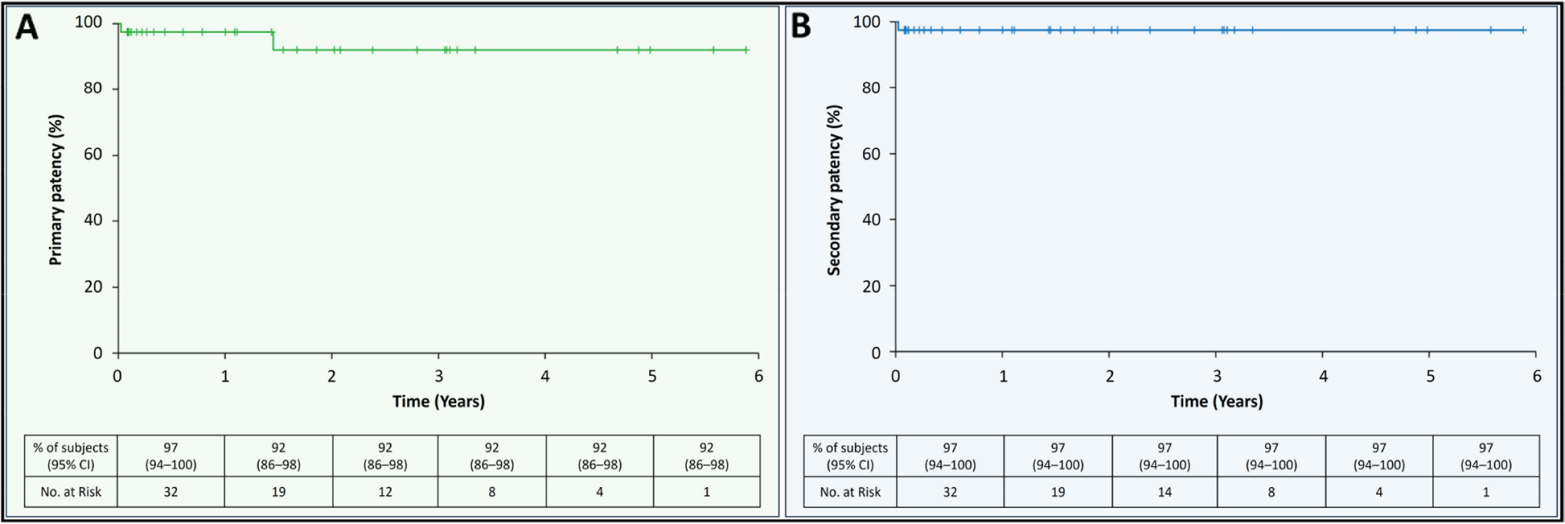
Long-term patency rates for Viabahn stent graft treating vessel injury. Curves show patency rates (%) over 6 years in patients treated with Viabahn stent graft for vessel injury: **A** primary patency (green curve), and (**B**) secondary patency (blue curve). CI = confidence interval

Median follow-up was 402 days [IQR, 43–1093], ranging from 9 to 2147 days. Kaplan–Meier analysis revealed primary patency rates of 97% (95% CI: 94–100) at year 1, and 92% (95% CI: 86–98) at year 6 following the Viabahn procedure, Fig. 1. One stent graft occlusion in the external iliac artery occurred after 18 months; endovascular revascularization was successful. Subsequently, secondary patency rates remained at 97% (95% CI: 94–100) at both year 1 and year 6. No major or minor amputations were reported.

## Discussion

In this study, we evaluated the performance of Viabahn stent grafts in emergency endovascular treatment (EVT) for acute arterial injury at various vessel sites. The implantation of a Viabahn endoprosthesis is safe, as there were no intraoperative adverse events, and effective, underlined by technical and clinical success rates of 100%. The 30-day major adverse event (MAE) rate was 2.5%. Long-term outcomes were also favorable, with a primary patency rate of 97% (95% CI: 94–100) in year 1 and 92% (95% CI: 86–98) from years 2 to 6. Secondary patency rates remained consistent at 97% (95% CI: 94–100) from years 1 to 6.

**Fig. 2:**
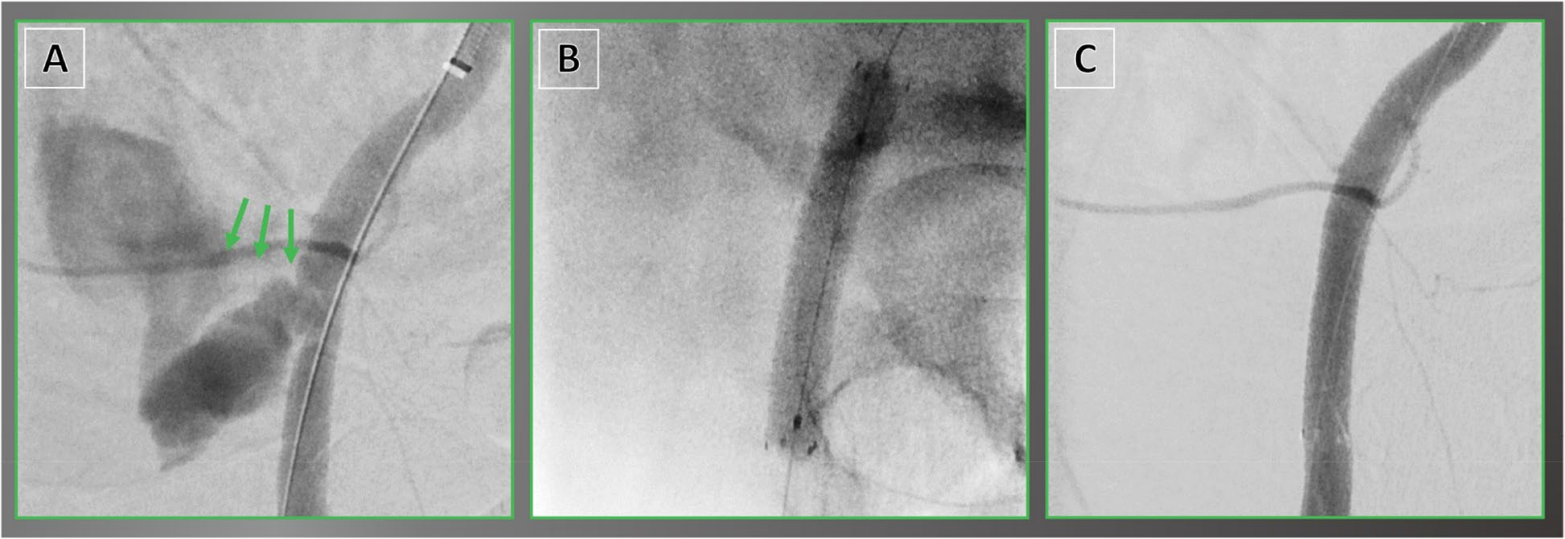
Viabahn stent graft for endovascular treatment of extravasation. A 79-year-old female with (**A**) fulminant bleeding (green arrows) from the common femoral artery after transcatheter aortic valve replacement procedure. **B** Implantation of a Viabahn stent graft after crossover maneuver. **C** Final target site angiography demonstrated successful hemostasis

**Fig. 3:**
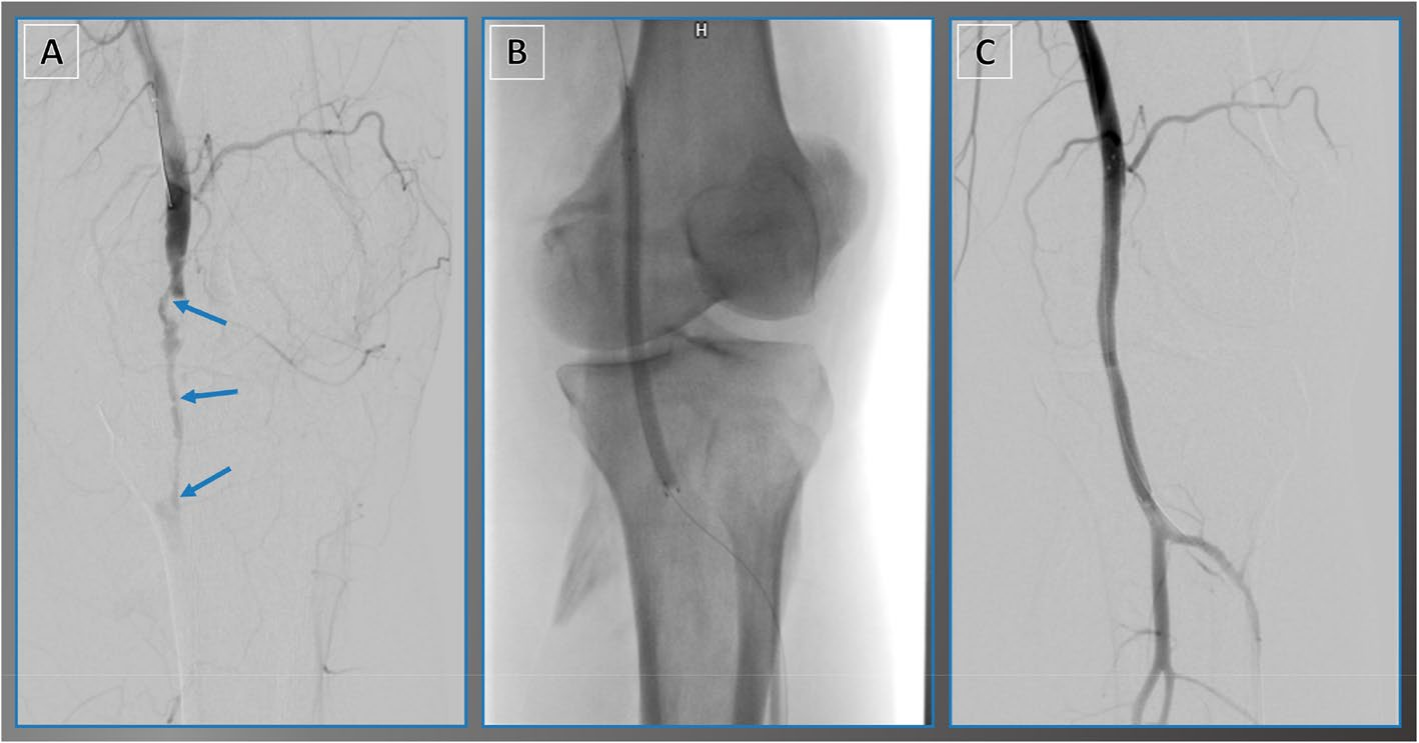
Viabahn stent graft for endovascular rupture treatment. A 52-year-old male reported experiencing leg pain following a work-related accident at a construction site. **A** Angiogram revealed traumatic rupture of the popliteal artery (blue arrows). **B** A Viabahn stent graft was deployed. **C** Final target site angiography demonstrated restored blood flow

**Fig. 4:**
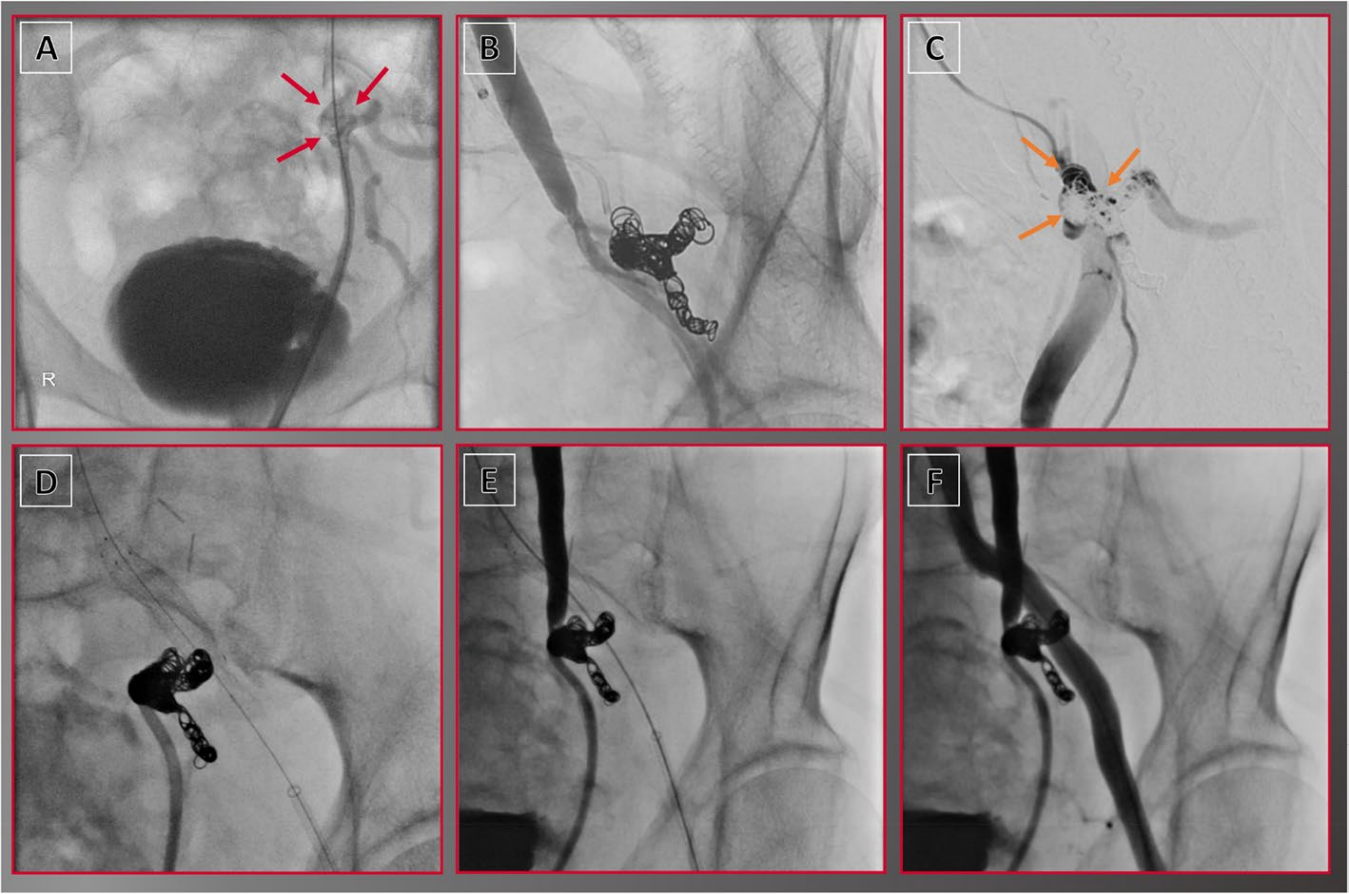
Viabahn stent graft for endovascular treatment of uretero-iliacal fistula. In an 80-year-old male patient with a complex medical history, including extensive prior surgery and chemotherapy for colon carcinoma, multiple prior surgeries for urolithiasis, and ureteric stent implantation, the removal of the ureteric stent led to massive hematuria. **A** Subsequent ureterorenoscopy revealed the presence of a fistulous connection between the left ureter and the adjacent left internal iliac artery (red arrows), **B** which was first addressed through coiling. **C** Due to residual perfusion (orange arrows), **D** a Viabahn stent graft was deployed. **E** Ureterography showed no remaining perfusion of the fistula. **F** Final target site angiography demonstrated preserved left iliac blood flow

Stent grafts have emerged as a valuable option for managing bleeding vessels when surgical intervention is either unsuitable or poses significant risks. In contrast to alternative endovascular techniques like coil or glue embolization, stent graft deployment has the potential to preserve the target vessel. The procedures in our study had a technical and clinical success rate of 100%, in line with previously reported rates of 97 to 100% [9, 10, 13]. We did not observe any intraoperative complications, possibly because of the stent graft's flexibility, which aids in navigating through convoluted arteries and maintaining flow at points of flexion, and the lower radial forces exerted on the damaged arterial wall when employing the self-expanding Viabahn stent graft compared to balloon-expandable stent grafts, which in contrast have shown to perform better in calcified areas [14, 15]. With a 30-day MAE rate of 2.5%, the study contributes to the increasing body of evidence reinforcing the safety of the device for a range of vascular pathologies, such as stenosis, vessel injury, and aneurysm [16–19]. Even in EVT of arterial injury, periprocedural anticoagulation therapy can be carried out during stent graft procedures to prevent thrombus formation [20]. However, it is essential to ensure successful placement of the stent graft and evaluate the patient's hemodynamic stability. Priority should be given to effective management of bleeding, especially in cases where patients show signs of hemodynamic instability. Our study demonstrates good long-term outcomes with primary patency rates of 92% and secondary patency rates of 97% at 36 months. This is in accordance with previously reported primary patency rates of up to 100%, such as in vascular access complications during transcatheter aortic valve replacement or after percutaneous coronary interventions [21–24]. This provides reassurance that Viabahn endoprostheses have long-term durability and effectiveness in managing access site bleeding in these particular clinical scenarios, Fig. 2. Moreover, the Viabahn stent graft has proven to be a beneficial choice for endovascular repair in peripheral artery rupture by sealing off the entry tear and redirecting the flow of blood [25], Fig. 3. In our study, arteriovenous fistula held the second most common indication for EVT. It usually occurs due to trauma, but it can also develop from aneurysmatic segments or manifest congenitally [26, 27]. The options for treating arteriovenous fistulas remain a matter of debate [28]. One patient presented with uretero-iliacal fistula, Fig. 4. An endovascular approach using the Viabahn stent graft was chosen and resulted in both technical and clinical success. This favorable outcome is particularly remarkable considering the limited data on the utility of Viabahn stent graft for this rare indication [29]. Furthermore, the success in this case emphasizes the significance of endovascular techniques and highlights the usefulness of the Viabahn stent graft in challenging vessel injury conditions. Our study emphasizes the suitability of minimally invasive endovascular techniques for specific patient populations, providing distinct advantages for those with comorbidities or complex anatomical features that necessitate less invasive options. Although open repair plays a crucial role in treating vascular injuries, it is important to address potential drawbacks such as access-related trauma and subsequent complications like delayed wound healing, lymphatic fistulas, or prolonged hospital stays. These issues are especially problematic for elderly and multimorbid patients. Minimally invasive stent graft procedures seem to present a promising alternative, offering durable arterial reconstruction. Choosing the optimal approach requires a comprehensive assessment of patient factors, lesion characteristics, and long-term goals for success while minimizing procedural risks. Therefore, an essential aspect of promoting patient care involves utilizing a collaborative, interdisciplinary decision-making process that integrates the expertise of open surgical and interventional specialists.

This study has several limitations. First, while the data for this study are from a prospective database, it is important to note that the study itself constitutes a retrospective, single-arm analysis and lacks a control group. Second, the study examined a patient population from a single German tertiary center, which limits its generalizability across centers and countries. Third, identification and management of confounding variables may be hampered by the all-comers setting chosen.

## Conclusions

The use of Viabahn stent graft in emergency endovascular therapy for arterial injuries demonstrated technical and clinical success rates of 100%, a low 30-day major adverse event rate of 2.5%, and excellent long-term primary and secondary patency rates. Further research is warranted (*i*) to assess factors that impact both immediate and long-term outcomes, and (*ii*) to determine which patients benefit most from endovascular treatment compared to open surgical vascular repair.

## Data Availability

The datasets used and/or analysed during the current study are available from the corresponding author on reasonable request.

## Abbreviations

cdTLR: Clinically driven target lesion revascularization
EVT: Endovascular treatment
IDDM: Insulin-dependent diabetes mellitus;
MAE: Major adverse events
